# The layered crisis of the primary care medical workforce in the European region: what evidence do we need to identify causes and solutions?

**DOI:** 10.1186/s12960-023-00842-4

**Published:** 2023-07-14

**Authors:** Giuliano Russo, Julian Perelman, Tomas Zapata, Milena Šantrić-Milićević

**Affiliations:** 1 Wolfson Institute of Population Health, Queen Mary University of London, London, United Kingdom; 2 Nova National School of Public Health and Comprehensive Health Research Center, Nova University of Lisbon, Lisbon, Portugal; 3 Division of Country Health Policies and Systems, WHO Regional Office for Europe, Copenhagen, Denmark; 4 Faculty of Medicine, School of Public Health and Health Management, Institute of Social Medicine, University of Belgrade, Belgrade, Serbia

## Abstract

Primary care services are key to population health and for the efficient and equitable organisation of national health systems. This is why they are often financed through public funds. Primary care doctors are instrumental for the delivery of preventive services, continuity of care, and for the referral of patients through the system. These cadres are also the single largest health expenditure at the core of such services. Although recruitment and retention of primary care doctors have always been challenging, shortages are now exacerbated by higher demand for services from aging populations, increased burden of chronic diseases, backlogs from the COVID-19 pandemic, and patient expectations. At the same time, the supply of primary care physicians is constrained by rising retirement rates, internal and external migration, worsening working conditions, budget cuts, and increased burnout. Misalignment between national education sectors and labour markets is becoming apparent, compounding staff shortages and maldistribution. With their predominantly publicly funded health systems and in the aftermath of COVID-19, countries of the European region appear to be now on the cusp of a multi-layered, slow-burning primary care crisis, with almost every country reporting long waiting lists for doctor appointments, shortages of physicians, unfilled vacancies, and consequently, added pressures on hospitals’ Accident and Emergency services. This articles collection aims at pulling together the evidence from countries of the European Region on root causes of such workforce crisis, impacts, and effectiveness of existing policies to mitigate it. Original research is needed, offering analysis and fresh insights into the primary care medical workforce crisis in wider Europe. Ultimately, the aim of this articles collection is to provide an evidence basis for the identification of policy solutions to present and future primary health care crises in high as well as lower-income countries.

## Introduction

Primary health care (PHC) has long been recognised as key for population health for its vision of disease prevention, health promotion and social determinants of health, as well as its focus on rehabilitative, mental health, maternal and child health care services, and non-communicable diseases [1]. Strong primary care services are essential for efficient healthcare systems, as they represent the first call of port for patients, resolving 90% of cases or routing them towards suitable specialised levels of care. It is therefore no surprise they are considered instrumental for the achievement of Universal Health Coverage and Sustainable Development Goals worldwide [2].

Primary care physicians—also known in some countries as general practitioners (GPs) or family doctors—are essential for the organisation and provision of such services, providing diagnoses, advice, and continuity of care for patient-centred systems. Such cadres represent the largest expenditure at the core of PHC services, and act as gatekeepers for important national healthcare resources. Because its long-term dividends accrue to the wider population and its key role for equitable access to care, PHC in many countries is financed primarily through public funds [3].

Perhaps because of their welfare states and publicly funded healthcare models, European countries have traditionally fared better in the provision of PHC services than their privately funded counterparts in other high-income nations [4]. However, there are now signs that positions are shifting across different countries in the European region, such as patients in the UK struggling to get GP appointments and their government “…rising the alarm on the future of general practice” [5], the French media pondering why “…Europe is running out of doctors” [6], and the World Health Organization calling the current physicians shortage “…a ticking bomb in the European region” [7]. Many are the causes at the heart of such crisis, and their patterns and implications differ across the very diverse European region.

## The possible drivers of the crisis

Recruiting and retaining PHC doctors has historically been complex, as general practice is by some considered less attractive than other medical specialties. It often entails rural deployment and worse working conditions, higher burn-out rates, comparatively lower pay and prestige [8]. However, new demand as well as supply-side factors seem to be rocking the medical labour market in Europe, particularly in the aftermath of the COVID-19 pandemic.

Europe’s aging population, with its increased burden of multimorbidity and non-communicable diseases, the backlog and consequences of unseen cases from the pandemic lockdowns, and perhaps populations' compounded expectations for care, have put additional pressures on systems and frontline workers [9].

A recent survey of health workers showed there are currently 8.93 primary care physicians per 10,000 people in the European region; despite a slight increase in the last decade, maldistribution implies that some countries are less benefitted than others [10]. National stocks of primary care doctors are dwindling because of the concomitant challenges of retaining existing cadres and recruiting new ones. Retaining such highly mobile, skilled and in high-demand professionals is now proving particularly hard, amid the perception that workloads in primary care practices have increased but working conditions have worsened after years of underinvestment [11]. In the pre-pandemic austerity years, health budgets shrank across the board in the UK, but the hospitals’ share of NHS funding actually increased at the expense of community care [12]. In countries like Portugal, primary care vacancies in the National Health Service go unfilled, with many family doctors opting for employment in the private sector [13]. In countries like Serbia, four-fifths of medical students declare their intent to migrate after graduation, pushed by widening salary differentials with Western Europe countries [14]. In the aftermath of joining the European Union, the outflow of medical professionals from Romania and Bulgaria have led to critical workforce shortages in the national health care systems [15]. Despite having one of the highest rates of physicians per population, Greece has also the smallest proportion of primary care doctors in Europe, lengthening waiting times for physician appointments, which is pushing patients towards hospitals’ Accident and Emergency departments or the private sector, where they may face out-of-pocket expenditures [16].

Training new doctors is expensive and takes time. For the last five decades, *numerus clausus* policies have capped the number of students in medical schools for most European countries, and favoured the selection of academically minded, but not necessarily PHC-oriented candidates [17]. As prospective specialists are all recruited from the same pool of qualified students, recruiting primary care physicians proves difficult because of the perceived diminishing attractiveness of PHC employment in comparison to other specialties. Misalignment is now becoming apparent between the education sector and health labour markets [18], as medical curricula are perceived to be biased towards hospital-based specialties, with little PHC training and field experience offered to perspective doctors [19]. The view is consolidating in some quarters that a ‘PHC fit-for-purpose training’ should be introduced in medical undergraduate curricula, with extra credits devoted to general/family medicine and community or rural health practice, covering supervised practical experience in primary care settings, and with teaching staff specialised in general practice [20].

## What evidence is needed to identify solutions?

Unsurprisingly, such perceived shortage of primary care physicians has attracted the attention of the media in Europe, and ignited speculations on what to do about it. As usual, throwing more cash at primary care appears to be the default policy suggestion [3]. Although extra funds for primary care must unquestionably play a part in the solution, such narrow view fails to recognise the different roots of the crisis in the very diverse countries of the European region,^1^ as well as the complexities of intervening in the continent’s medical labour market.

So, what evidence do we exactly need for policy-makers and health practitioners to take sensible action? First of all, documenting and measuring the extent of shortages and increased workloads in different PHC markets of the European region would help identify present as well as future priorities, and lay out an agenda for research.

Secondly, qualitative and quantitative evidence on likely causes of the medical workforce crises in different settings would be particularly welcome, as well as cross-country comparative studies, with a view to generating hypotheses on what is working in PHC in Europe, what has not, and for what reasons. Specific analysis of European health labour markets would be particularly welcome, given the increased recognition that supply and demand forces are now receiving in the human resources for health area [21]. Finally, documenting what is currently being done to mitigate the effects in different quarters is sorely needed, together with convincing evidence on what interventions appear to have worked, for what countries, and under what circumstances.

Ultimately, this article collection aims at providing an evidence-based platform for academics to identify a research agenda on PHC, for policy-makers to identify suitable solutions, and for health practitioners to ponder upon their employment, recruitment and management decisions.

## Data Availability

Not applicable.

## Footnotes

1 See a list of such European region countries as defined by the WHO at: https://www.who.int/europe/about-us/about-who-europe.
